# Is there any radiation-induced brachial plexopathy after hypofractionated postmastectomy radiotherapy with helical tomotherapy?

**DOI:** 10.3389/fonc.2024.1392313

**Published:** 2024-04-29

**Authors:** Thinnakorn Chomchai, Pitchayaponne Klunklin, Siam Tongprasert, Thanat Kanthawang, Piyapasara Toapichattrakul, Imjai Chitapanarux

**Affiliations:** ^1^ Division of Radiation Oncology, Department of Radiology, Faculty of Medicine, Chiang Mai University, Chiang Mai, Thailand; ^2^ Department of Rehabilitation Medicine, Faculty of Medicine, Chiang Mai University, Chiang Mai, Thailand; ^3^ Department of Radiology, Faculty of Medicine, Chiang Mai University, Chiang Mai, Thailand

**Keywords:** radiation induced brachial plexopathy (RIBP), hypofractionated postmastectomy radiotherapy, breast cancer, helical tomotherapy (HT), intensity-modulated radiation therapy (IMRT)

## Abstract

**Introduction:**

Radiation-induced brachial plexopathy (RIBP) is one of the most concerning late radiation effects after hypofractionated postmastectomy radiotherapy (HF-PMRT) to the chest wall and regional lymph nodes. The purpose of this study was to investigate the RIBP events occurring in breast cancer patients after HF-PMRT using intensity-modulated radiation therapy (IMRT) by helical tomotherapy. Furthermore, the dosimetric parameters of the ipsilateral brachial plexus were reported.

**Materials and methods:**

Breast cancer patients who underwent HF-PMRT using the IMRT via HT at our institute were included. In the first cohort, subjective RIBP symptoms were measured using a QuickDASH questionnaire, whereas objective RIBP events were assessed using a comprehensive physical evaluation in the second cohort. The ipsilateral brachial plexus from all eligible patients’ treatment plans was contoured, and the dosimetric parameters were explored.

**Results:**

From March 2014 to December 2022, 229 patients were enrolled; 107 and 72 individuals were in the first and second cohorts, respectively. The first cohort’s median follow-up period was 27 months, and the second cohort was 31 months. In the first cohort, 80 patients (74.77%) had a normal function, 21 (19.63%) had a mild grade, and 6 (5.61%) had a moderate grade; no severe or very severe RIBP was observed. However, the comprehensive physical evaluation of the second cohort indicated no RIBP events. Dosimetric analysis revealed that the median maximum dose was 44.52, 44.52, and 44.60 Gy; the median mean dose was 33.00, 32.23, and 32.33 Gy; and the median dose at 0.03 cc was 44.33, 44.36, and 44.39 Gy for all patients, patients in the first and second cohort, respectively. Each dosimetric parameter was evaluated, and no statistically significant differences were detected.

**Conclusion:**

The absence of RIBP events supports the safety of employing HF-PMRT by HT for the chest wall and all regional lymph nodes. We propose that applying the ICRU Report 83 criteria for IMRT planning, which limit the maximum dose (107% of the prescribed dose) to less than 2% of the planning target volume and exclude the brachial plexus region from the maximal dose area, is a practical way to minimize the risk of RIBP from HF-PMRT.

## Introduction

Numerous research studies have established that adjuvant postmastectomy radiotherapy (PMRT) to the chest wall and regional lymph nodes offers significant advantages in terms of overall survival (OS) and locoregional control (LRC) compared to patients who only receive adjuvant chemotherapy following mastectomy (1–4). Consequently, PMRT is considered the standard of care for breast cancer patients with T3-4 tumors, regional nodal involvement, or a positive surgical margin after mastectomy. In addition, the adoption of the hypofractionated (HF) regimen for PMRT is growing in popularity because it provides similar outcomes regarding OS, LRC, and toxicities to the conventional fractionated (CF) regimen (5–7).

Presently, the application of HF-PMRT is widespread, including within our institute. However, given concerns about potential adverse effects, the National Comprehensive Cancer Network (NCCN) guidelines still recommend the HF regimen as an alternative to the CF regimen for PMRT (8). Radiation-induced brachial plexopathy (RIBP) is one of the concerning long-term adverse effects of PMRT. Since the brachial nerve plexus is usually included in high dose treatment volumes, it receives an 18-20% greater radiation dose than the prescribed isodose (9). Several radiation therapy factors may further increase the risk of radiation-induced brachial plexopathy (RIBP), including the radiation technique, dose fractionation, and specific treatment sites such as the axillary regions. This vulnerability is produced by both direct damage to neurons or glial cells and indirect effects of ischemic damage resulting from microvascular injury or stenosis due to the late radiation complication (10). Even though the total reported of RIBP has decreased over time due to advances in radiation techniques, it is still regarded as an important adverse effect of HF-PMRT. This is because RIBP can result in an irreversible condition that significantly reduces individuals’ quality of life (11).

An earlier large retrospective study examining the rate of RIBP in HF-PMRT utilizing conventional (2D) and 3-dimension conformal therapy (3D-CRT) techniques revealed a wide range of 1.7% to 73% (12). We were only able to locate one publication that documented a minimal rate of RIBP (0.4%) subsequent to CF-PMRT employing intensity-modulated radiation therapy (IMRT) (13); therefore, there is currently a significant lack of data about RIBP after HF-PMRT with IMRT. Nonetheless, to the best of our knowledge, no published information regarding RIBP following HF-PMRT with IMRT given by helical tomotherapy (HT) is available. As a different method of IMRT provided by HT and with our long experience of employing HF-PMRT via HT since 2014, this study aims to describe the RIBP event from HF-PMRT with the IMRT technique via HT as well as investigate the dosimetric factors associated with RIBP.

## Materials and methods

### Patient selection

Following ethical approval from our institutional review board (Research ID: 9111), we evaluated the medical records of breast cancer patients who underwent HF-PMRT by IMRT technique via HT at our center from March 2014 to December 2022. The study comprised 18 to 70-year-old patients who were treated with curative intent and had completed radiotherapy at least 6 months before clinical evaluation. Adjuvant or neoadjuvant hormonal, targeted, or chemotherapy treatments were allowed. Patients with pre-existing brachial plexus impairment, a history of brachial plexus radiation, palliative intent, locoregional recurrence, or distant metastases were excluded.

In accordance with our institute’s treatment approach, all patients in this study received PMRT to the chest wall, including the surgical scar, supraclavicular, axillary level I-III, and internal mammary lymph node areas. The HF-PMRT was delivered by HT machine with a field width of 2.5 or 5 centimeters, a pitch of 0.287 or 0.215, and a modulation factor ranging from 2.5 to 3.5. A HF regimen was employed, with a dose of 2.65-2.67 Gy per fraction daily, for a total of 15-16 fractions while in cases of positive margins or T4d disease, 18 fractions were administered. Treatment targets were evaluated using three specific criteria from the International Commission on Radiation Units and Measurements (ICRU) report 83:

1. 95% of the prescribed dose covered at least 98% of the planning target volume (PTV).
2. The prescribed dose covered at least 50% of the PTV.
3. 107% of the prescribed dose covered less than 2% of the PTV.

### Radiation-induced brachial plexopathy assessment

The RIBP events were documented in two cohorts. The first cohort was assessed using a QuickDASH questionnaire (14), which was created to subjectively assess symptoms and the ability to do tasks as described in the Hand, Shoulder, and Arm Disabilities. Conversely, the search for RIBP by the second cohort involved a comprehensive physical evaluation that included a complete physical examination and rigorous diagnostic modalities.

Initially, all eligible patients were contacted via telephone or postal mail to inform them of this study. Subsequently, they were scheduled to visit our clinic to complete the QuickDASH questionnaire and undergo a comprehensive evaluation. Patients who were unable to attend the survey in person completed it via phone calls. The QuickDASH scores were calculated and reported following a subjective RIBP classification system (normal, mild, moderate, severe, and very severe).

Upon the completion of the QuickDASH questionnaire, visiting patients who accepted to participate in the second cohort underwent physical examination by a radiation oncologist and a physiatrist (rehabilitation medicine specialist) following the guidelines specified in the Guides to the Evaluation of Permanent Impairment (15) published by the Social Security Office of Thailand. The complete physical examination included testing the motor power and sensory function of the brachial plexus. The results were then interpreted and classified as a grading system for motor and sensory impairments (Grade 0–4). In addition, nerve conduction study (NCS), electromyography (EMG), and magnetic resonance imaging (MRI) were used to confirm the diagnosis of RIBP in people who were thought to be showing signs and symptoms of the condition.

### Dosimetric assessment of brachial plexus

The ipsilateral brachial plexus of treatment planning for all eligible patients had been contoured by a radiation oncologist according to the RTOG-Endorsed Brachial Plexus Contouring Atlas (16). Later, a single musculoskeletal imaging diagnostic radiologist reviewed the brachial plexus contours to confirm adherence to standard contouring guideline. Following the contouring process, the essential dosimetric parameters of the brachial plexus, including the maximum dose, mean dose, D2cc, and D0.03cc, were determined from real treatment planning data. The linear quadratic method was used to convert the dosimetric data from the brachial plexus to its equivalent dose in 2 Gy fractions (EQD2). The formula assumed that the brachial plexus had an a/b of 3 (17). To guarantee consistency, the dosimetric data of all eligible patients and patients in both cohorts were also evaluated and compared.

### Study endpoints and statistical analysis

Patient characteristics of eligible patients and patients in both cohort groups were presented through descriptive statistics as mean or median accompanied by standard deviation (SD) or interquartile range (IQR). The rate and severity of RIBP resulting from the QuickDASH questionnaire (the first cohort) and the comprehensive physical evaluation (the second cohort) were reported as a percentage. All eligible patients’ brachial plexus dosimetric parameters, as well as those of the patients in both cohorts, were detailed in the descriptive analysis and compared using the Kruskal-Wallis H test. A P-value of less than 0.05 was considered a statistically significant difference in the analysis. All statistical analysis was performed using SPSS statistical software (version 16.6, SPSS Inc., 444 N. Michigan, Chicago, IL, USA).

## Results

**Figure 1:**
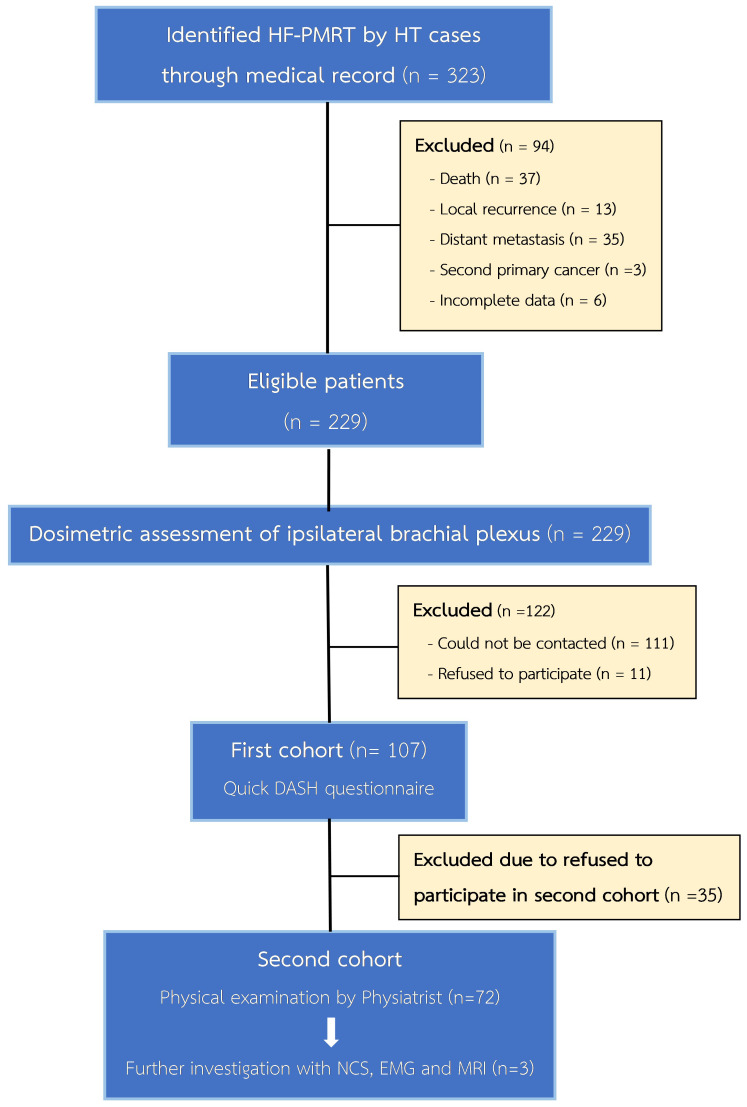
Consort diagram.

A total of 229 patients met the inclusion criteria for our study; all their treatment plans were examined for ipsilateral brachial plexus dosimetry. In the first cohort for RIBP assessment, 107 patients were included to be evaluated via the QuickDASH questionnaire. In the second cohort, 72 out of the 107 patients were subjected to our comprehensive physical evaluation, as shown in **Figure 1**. Details regarding the baseline patients and treatments’ characteristics can be found in **Table 1**. All of these patients underwent a modified radical mastectomy, which involves removing the entire breast, including the skin, areola, and nipple, as well as dissecting axillary lymph nodes at levels I and II. Almost all patients (98.69%) received chemotherapy, and the regimens applied were also listed in **Table 1**. In addition, hormone therapy was given to 82.97% of patients with hormone receptor-positive malignancies. The median follow-up time for the first cohort was 27 months (IQR 15-76 months), while for the second cohort it was 31 months (IQR 19-83 months). A median radiation dose to the chest wall of 42.4 Gy (range 40.05–47.7 Gy) and RNI of 42.4 Gy (range 40.05–42.4 Gy) were prescribed. These doses were administered in fractions ranging from 15 to 18 fractions.

**Table 1:** Baseline characteristics.

Variables	All patients (n= 229)	First cohort (n=107)	Second cohort (n=72)
**Follow-up time** (Median; IQR) (month)	–	27 (15-76)	31 (19-83)
**Age** (Mean; SD) (year)	60.62 (10.16)	60.43 (10.19)	59.50 (9.54)
**Tumor size** (Median; IQR) (cm)	3.20 (2.50-4.50)	3.50 (2.50-4.50)	3.35 (2.40-4.85)
**Nodal involvement** (Median; IQR) - Number of nodal positive - Number of nodal dissected - Nodal ratio	4 (2-7) 12 (8-17) 0.33 (0.17-0.67)	4 (1-7) 11 (7-16) 0.35 (0.18-0.69)	4 (2-7) 12 (8-18) 0.35 (0.20-0.68)
**AJCC stage (N; %)** - II - III	74 (32.31) 155 (67.69)	33 (30.84) 74 (69.16)	19 (26.39) 53 (73.61)
**Chemotherapy (N; %)** - No - Yes + AC + FAC + AC then Taxane + TC	3 (1.31) 226 (98.69) 2 (0.87) 8 (3.49) 215 (93.89) 1 (0.44)	1 (0.93) 106 (99.07) 2 (1.87) 8 (7.48) 95 (88.79) 1 (0.93)	0 (0) 72 (100) 2 (2.78) 6 (8.33) 63 (87.50) 1 (1.39)
**Hormonal therapy (N; %)** - No - Yes	39 (17.03) 190 (82.97)	40 (37.38) 67 (62.62)	26 (36.11) 46 (63.89)
**Prescribed dose** **Chest wall (Median; range) (Gy)** - 40.05 Gy/15 Fx (N; %) - 42.40 Gy/16 Fx (N; %) - 47.70 Gy/18 Fx (N; %) **Regional nodes (Median; range) (Gy)** - 40.05 Gy/15 Fx (N; %) - 42.40 Gy/16 Fx (N; %)	42.40 (40.05-47.70) 8 (3.49) 193 (84.28) 28 (12.23) 42.40 (40.05-42.40) 8(3.49) 221(96.51)	42.40 (40.05-47.70) 2 (1.87) 95 (88.79) 10 (9.34) 42.40 (40.05-42.40) 2(1.87) 105(98.13)	42.40 (40.05-47.70) 2 (2.78) 64 (88.89) 6 (8.33) 42.40(40.05-42.40) 2(2.78) 70(97.22)

IQR, interquartile range; SD, Standard deviation; AJCC, American Joint Commission on Cancer; A, Adriamycin; C, Cyclophosphamide; F; Fluorouracil.

**Figure 2:**
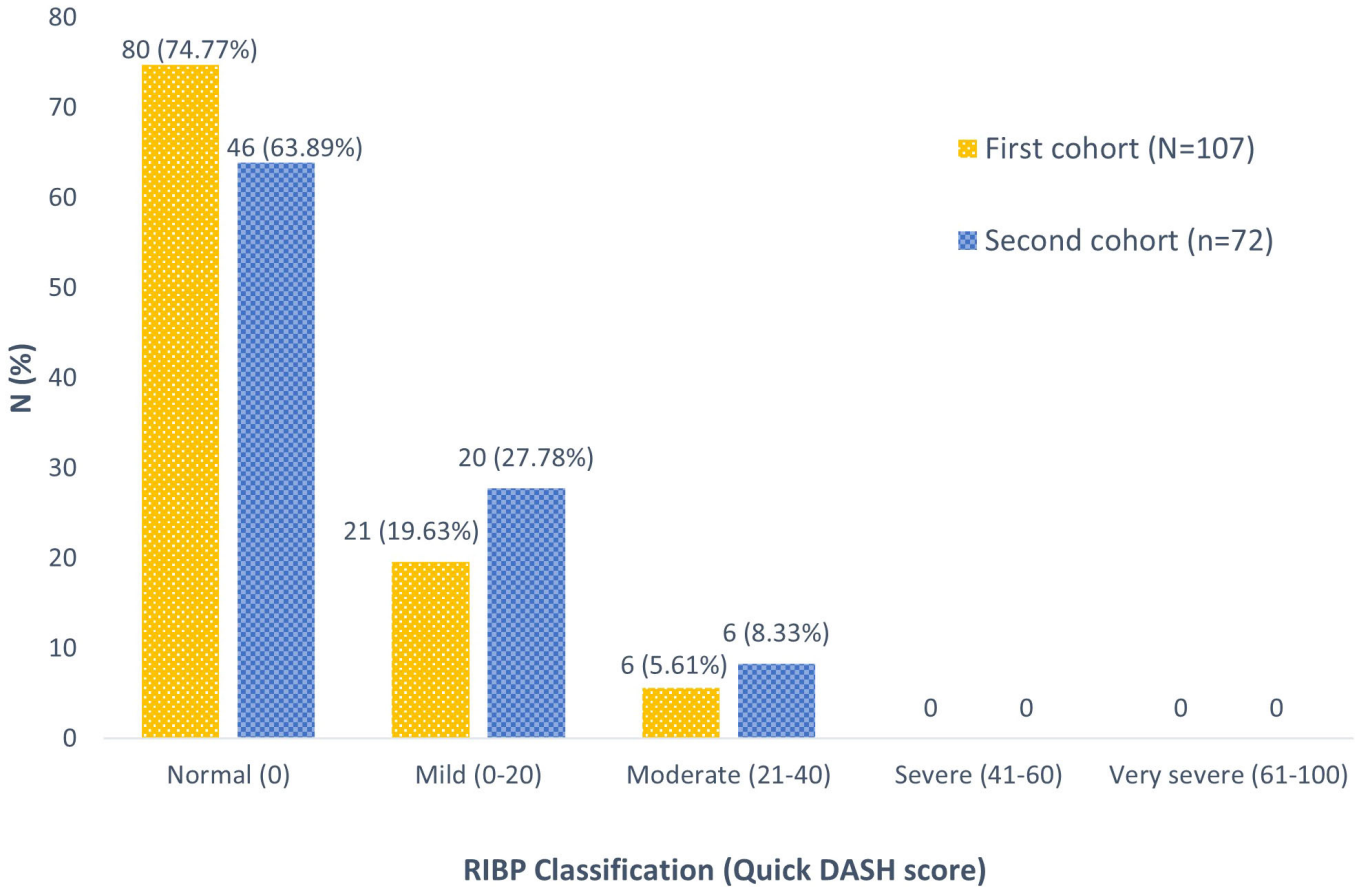
The RIBP classification based on QuickDASH score.

From the first cohort, subjective outcome evaluations based on QuickDASH questionnaire scores were presented as RIBP classifications (**Figure 2**). Most patients (74.77%) exhibited normal brachial plexus function. Additionally, 21 patients (19.63%) reported a mild grade, while 6 patients (5.61%) experienced a moderate grade of RIBP. Notably, no cases of severe or very severe RIBP in the ipsilateral arm were reported within this cohort.

Seventy-two patients continued to the second cohort. Additionally, except for one patient with mild grade RIBP, practically 26 patients who reported subjective RIBP symptoms in the first cohort carried out this comprehensive physical evaluation. Upon a physical examination, three patients (4.17%) presented clinical signs and symptoms similar to brachial plexopathy, such as sensory abnormalities and diminished hand function (**Table 2**). Diagnostic procedures for brachial plexopathy, including NCS, EMG, and MRI, were performed on these 3 patients. Finally, the first patient’s NCS was found to be normal. The second patient was diagnosed with median nerve neuropathy in the wrist (PMRT side), which is also known as carpal tunnel syndrome, and the third patient was identified with sensory neuropathy, median neuropathy in both wrists, and suspected left (PMRT side) superficial radial neuropathy. Surprisingly, no patients showed genuine evidence of motor and sensory deficits of brachial plexus origin, reflecting that there were no recorded events of RIBP discovered throughout our study.

**Table 2:** The motor and sensory impairment classification from physical examination in second cohort base on the Guides to the Evaluation of Permanent Impairment (15).

Classification	Motor deficit (N=72) N (%)	Sensory deficit (N=72) N (%)
0	72 (100)	69 (95.8)
1	0 (0)	3 (4.2)
2	0 (0)	0 (0)
3	0 (0)	0 (0)
4	0 (0)	0 (0)

In the dosimetric analysis, the median volume of the ipsilateral brachial plexus was 4.82 cc (range 2.97-8.63 cc) in all patients, 4.76 cc (range 3.36-8.63 cc) in the first cohort, and 4.82 cc (range 3.36-8.63 cc) in the second cohort. Each dosimetric parameter of the ipsilateral brachial plexus from three patient groups was investigated and presented in **Table 3**. The median maximum dose was 44.52, 44.52, and 44.60 Gy; the median mean dose was 33.00, 32.23, and 32.33 Gy; and the median dose at 0.03 cc (D0.03cc) was 44.33, 44.36, and 44.39 Gy for all patients, the first and second cohorts, respectively. Each parameter was compared after the dosage had been converted to EQD2 using the α/β of 3, and no statistically significant difference was observed.

**Table 3:** Hypofractionated dose and EQD2 of the ipsilateral brachial plexus.

Dosimetric Parameters	All (n=229)		First cohort (n=107)		Second cohort (n=72)		P-value (EQD2)
	Hypo- fractionated dose	EQD2	Hypo- fractionated dose	EQD2	Hypo- fractioned dose	EQD2	
**Maximum dose (Gy)** - Range - Median	34.44-50.61 44.52	36.48-58.83 51.39	41.71-50.01 44.52	47.57-57.80 51.42	41.71-50.01 44.60	48.23-57.80 51.60	0.79
**Mean dose (Gy)** - Range - Median	4.96-45.94 33.00	3.31-51.02 33.22	15.39-41.30 32.23	12.20-46.11 31.44	15.39-41.30 32.33	12.20-46.11 32.61	0.81
**D2cc (Gy)** - Range - Median	29.85-50.06 44.11	29.80-57.89 50.69	41.20-49.39 44.08	45.94-56.74 50.59	41.20-49.39 44.15	45.94-56.74 50.75	0.81
**D0.03cc (Gy)** - Range - Median	32.33-50.12 44.33	33.34-58.87 51.10	41.39-49.77 44.36	46.33-58.87 51.13	41.39-49.77 44.39	46.33-57.39 51.24	0.89

## Discussion

As previously mentioned, RIBP, in spite of its low reported rate, is one of the considered adverse effects of PMRT. Lymph node irradiation in PMRT, particularly in the axillary and SPC regions, has an influence on the development of RIBP since the radiation field always includes some portion of the brachial plexus. Furthermore, the HF-PMRT regimen increases the risk of RIBP as the brachial plexus is a serial organ and late-responding tissue that responds to larger doses per fraction (18). Therefore, our study focused on the effect of radiation on brachial plexopathy development and determined the RIBP event in patients receiving HF-PMRT using the IMRT technique via HT machine. To the best of our knowledge, this is the first study to report on RIBP events and branchial plexus dosimetry from HF-PMRT by HT.

Interestingly, from this cohort analysis, none of our patients developed RIBP after undergoing HF-PMRT utilizing the IMRT approach with HT. Although 25.23% of individuals were classified as having clinical brachial plexopathy based on the QuickDASH score, no one was confirmed to have RIBP after a comprehensive physical evaluation in the second cohort. Historically, HF-PMRT (2.2-4.58 Gy/Fx to 36-60 Gy) with the 2D technique resulted in a varying rate of RIBP, ranging from 1.7% to 73%, with a median follow-up time of 60-408 months (10, 12, 19, 20). A prior study from our center discovered that approximately 2% of patients who received HF-PMRT using 2D/3D-CRT techniques experienced RIBP (6). This is consistent with more recent studies, which reported no cases of brachial plexopathy (5, 7).

There is only one report on RIBP from the IMRT technique, which was conducted using CF-PMRT. Rudra et al. (13) found a low RIBP rate of 0.4% among 258 patients who received radiation of 50-50.4 Gy (1.8-2 Gy/fraction) to the chest wall, internal mammary, axillary, and supraclavicular lymph nodes with IMRT.

It is established that RIBP typically manifests as a long-term side effect. According to Johansson et al. (10), the median time for brachial plexopathy symptom development using the 3D-CRT approach ranged from 2 to 7 years, whereas the median time to develop RIBP with the IMRT technique (13) was 45 months (range, 19-127 months). In our current study, the median follow-up length was 31 months (IQR 19-83), with the longest follow-up period reaching 112 months, which could be long enough to identify RIBP. It is unclear if this period is sufficient to cover the follow-up period for the RIBP rate after HF-PMRT by HT, but continuous monitoring is critical for determining the development of RIBP over time.

In the 2D era, Galecki et al. (12) mentioned that the rate of RIBP was below 1% when the total administered dose ranged from 34 to 40 Gy for the HF regimen. The dosimetric data from Rudra et al. (13) indicated a median Dmax and Dmean for the brachial plexus of 54.8 Gy and 44.1 Gy, respectively, resulting in a low rate of RIBP from IMRT treatment planning with the CF regimen. Our institute’s protocol uses a maximum dose of less than 107% of the prescribed dose (40.05 Gy in 15 Fx, 42.40 Gy in 16 Fx, and 47.70 Gy in 18 Fx) to instruct treatment planning. Our dosimetric analysis of the brachial plexus indicated a median Dmax of 44.52 Gy (EQD2 51.39 Gy) and Dmean of 33 Gy (EQD2 33.22 Gy), which are lower than prior IMRT data, suggesting a potential influence on the observed no RIBP event in our study.

Other malignancies, including head and neck cancer and lung cancer, have also had reports of RIBP after radiotherapy. In head and neck cancer, with a median follow-up time of 16.2 months, Truong et al. (21) found no case of RIBP in 114 patients who underwent IMRT at a total dose of 69.96 Gy in 33 fractions with a brachial plexus mean Dmax of 58.1 Gy, and mean Dmean of 42.2 Gy. In lung cancer, Amini et al. (22) reported a 16% rate of RIBP in non-small cell lung cancer (NSCLC) patients after treatment with definitive chemoradiation, with a median follow-up time of 14.0 months. The median brachial plexus dose >69 Gy, the maximum dose >75 Gy to volume 2 cc of the brachial plexus, and the presence of plexopathy before radiation were identified as independent predictors of the plexopathy. Similarly, another report from NSCLC treatment observed RIBP in 5 out of 80 patients, suggesting a 3-year rate of 12%, and brachial plexus V76 ≥1 cc was identified as an influential factor for developing RIBP (23). These data from other cancer therapies reporting the low rate of RIBP and the extremely high dose to the brachial plexus as the contributing factors of RIBP support the safety of the brachial plexus while using HF-PMRT, which has a lower prescription dose than other cancer treatments.

Furthermore, our separate study of the brachial plexus dosimetry for each cohort group revealed no statistically significant difference from all dosimetric values (Dmax, Dmean, D2cc, and D0.03cc). These findings verified the coherence among all groups of patients, allowing us to infer that the RIBP event would be very low or not occur in all patients included in this study. On top of that, the diagnosis of RIBP in our study was highly reliable because the method comprised both a subjective evaluation from a QuickDASH questionnaire and an objective evaluation from a comprehensive physical evaluation, which consisted of a full physical examination by a physiatrist as well as additional investigations to confirm the RIBP diagnosis. In the first cohort, our study identified 21 patients (19.63%) with a mild grade and 6 patients (5.61%) with a moderate grade of RIBP using the QuickDASH classification. Generally, the QuickDASH questionnaire is used to assess the impact of a wide range of musculoskeletal disorders, traumas and lymphedema on upper extremity that are not limited to brachial plexopathy. As a result, after a comprehensive physical examination in the second cohort, it was discovered that all subjects exhibiting upper extremity impairment symptoms on the QuickDASH questionnaire were not diagnosed with RIBP. Additionally, 26 of the 27 patients (96.3%) who had clinical RIBP from the QuickDASH questionnaire underwent a comprehensive physical evaluation, which revealed that the RIBP did not exist. This could confirm that our first and second cohort groups did not indeed include any cases of RIBP. Lastly, our results demonstrated that a physical examination, even by a specialist, is insufficient to diagnose RIBP. Additional diagnostic procedures, such as an MRI, NCS, or EMG, are necessary for an accurate diagnosis of RIBP due to the considerable difference in treatment between RIBP and other neuromuscular conditions.

Given the limitations of our study, which was a cross-sectional investigation, the reporting of incidence may lack some ability to draw definite conclusions due to its focus on a particular point in time. Additionally, an underestimation of the true rate of RIBP might occur from examining only patients who have survived without local recurrence and metastasis, as those who have encountered RIBP may have been excluded. Owing to the absence of RIBP events, we also cannot identify any correlation between RIBP and the dosimetric parameters. Further prospective studies would be conducted to report a more accurate RIBP rate after HF-PMRT by HT and to have a better understanding of the potential risk factors associated with it. A larger sample size, including patients with various degrees of metastasis, would provide an accurate depiction of the true rate of RIBP. Furthermore, providing a pre-treatment assessment of brachial plexopathy as a baseline and continuing the trial over a longer period of time would allow monitoring and reporting of late-onset complications. As a whole, further study is needed to improve our understanding of RIBP and its effects on cancer survivors, which could facilitate better prevention, diagnosis, and treatment of RIBP.

## Conclusion

The absence of RIBP events from our study supports the safety of employing HF-PMRT by HT for the chest wall and all regional lymph nodes. We propose that applying the ICRU Report 83 criteria for IMRT planning, which limit the maximum dose (107% of the prescribed dose) to less than 2% of the PTV and exclude the brachial plexus region from the maximal dose area, is a practical way to minimize the risk of RIBP from HF-PMRT. However, additional prospective research is needed.

## Data availability statement

The raw data supporting the conclusions of this article will be made available by the authors, without undue reservation.

## Ethics statement

The studies involving humans were approved by The Research Ethics Committee of Faculty of Medicine, Chiang Mai University. The studies were conducted in accordance with the local legislation and institutional requirements. The participants provided their written informed consent to participate in this study.

## Author contributions

TC: Data curation, Investigation, Project administration, Writing – original draft. PK: Conceptualization, Funding acquisition, Methodology, Supervision, Writing – original draft, Writing – review & editing, Visualization. ST: Writing – review & editing, Investigation, Methodology, Validation. TK: Investigation, Validation, Writing – review & editing. PT: Validation, Writing – review & editing, Formal Analysis, Software. IC: Conceptualization, Supervision, Writing – review & editing.
